# High Mobility Group Box-1 Protein and Outcomes in Critically Ill Surgical Patients Requiring Open Abdominal Management

**DOI:** 10.1155/2017/6305387

**Published:** 2017-02-14

**Authors:** Michelle S. Malig, Craig N. Jenne, Chad G. Ball, Derek J. Roberts, Zhengwen Xiao, Andrew W. Kirkpatrick

**Affiliations:** ^1^Department of Microbiology, Immunology and Infectious Diseases, University of Calgary, Calgary, AB, Canada; ^2^Critical Care Medicine, University of Calgary, Calgary, AB, Canada; ^3^Department of General Surgery, University of Calgary, Calgary, AB, Canada; ^4^The Regional Trauma Program, University of Calgary, Calgary, AB, Canada

## Abstract

*Background*. Previous studies assessing various cytokines in the critically ill/injured have been uninformative in terms of translating to clinical care management. Animal abdominal sepsis work suggests that enhanced intraperitoneal (IP) clearance of Damage-Associated Molecular Patterns (DAMPs) improves outcome. Thus measuring the responses of DAMPs offers alternate potential insights and a representative DAMP, High Mobility Group Box-1 protein (HMGB-1), was considered. While IP biomediators are being recognized in critical illness/trauma, HMGB-1 behaviour has not been examined in open abdomen (OA) management.* Methods*. A modified protocol for HMGB-1 detection was used to examine plasma/IP fluid samples from 44 critically ill/injured OA patients enrolled in a randomized controlled trial comparing two negative pressure peritoneal therapies (NPPT): Active NPPT (ANPPT) and Barker's Vacuum Pack NPPT (BVP). Samples were collected and analyzed at the time of laparotomy and at 24 and 48 hours after.* Results*. There were no statistically significant differences in survivor versus nonsurvivor HMGB-1 plasma or IP concentrations at baseline, 24 hours, or 48 hours. However, plasma HMGB-1 levels tended to increase continuously in the BVP cohort.* Conclusions*. HMGB-1 appeared to behave differently between NPPT cohorts. Further studies are needed to elucidate the relationship of HMGB-1 and outcomes in septic/injured patients.

## 1. Introduction

Inflammatory biomediators enact diffuse end-organ damage in sepsis and the systemic inflammatory response syndrome (SIRS) but have been resistant to pharmacologic manipulation given their variable release, cross-reaction, and positive and negative feedback loops. Over one hundred attempts at blocking single biological response mediators have failed to regulate the early cytokine storm of sepsis [1]. The term Damage-Associated Molecular Patterns (DAMPs), a novel class of biomediators, describes molecules released by host cells upon lysis or injury, which signal for necrotic cell clearance by phagocytic cells of the immune system. Freely circulating DAMPs may trigger an inflammatory reaction, in much the same fashion as pathogen associated molecular patterns (PAMPs) found on many bacterial pathogens, by binding to host cell receptors on a variety of immune cells. Some DAMPs, such as High Mobility Group Box-1 protein (HMGB-1), have been shown to be both markers of damage and mediators of inflammation in sterile and nonsterile injury [2–4].

HMGB-1 is a nuclear protein involved in a number of transcriptional processes and, when released into the extracellular environment, HMGB-1 can stimulate and activate the immune system. Freely circulating HMGB-1 released passively from necrotic cells can activate macrophage via Toll-like receptor 4 (TLR-4) to stimulate cytokine production [2, 5, 6]. And with the release of cytokines, such as TNF*α* or IL-1, they further stimulate other macrophages to actively release HMGB-1 [3, 6], thus increasing the extracellular concentration of this DAMP and amplifying its response. In an animal sepsis model, using cecal ligation and puncture methods in mice, the administration of anti-HMGB-1 during the late stages improved survival [6]. Conversely, in the same study, administration of HMGB-1 to mice induced septic-like symptoms in the absence of any infectious agent. In a clinical study conducted by Gibot et al., it was found that disease severity correlated with elevated levels of HMGB-1 in late stage sepsis [7]; however, they also concluded that HMGB-1 concentrations could not be singly used as a marker for mortality among septic patients. Hence the role of HMGB-1 in sepsis and patient outcome remains unclear. These findings are further complicated by the discovery that HMGB-1 also has direct antimicrobial activity [8] and promotes neutrophil extracellular trap (NET) formation by associating with platelets [9]. These additional findings demonstrate that HMGB-1 is not only an inflammatory marker but also a potent driver of the innate immune response. Therefore, HMGB-1 levels may be a better marker of interest regarding those at risk of succumbing to the persistent inflammatory, immunosuppressive, catabolic response syndrome (PICS), which is increasingly important as septic patients survive their initial “cytokine storms” only to die later [1].

Open abdominal management involves leaving the midline abdominal fascia “open” to expedite and/or facilitate future relaparotomy [10] in an attempt to improve the outcomes of critically ill or injured patients [11–14]. In the interval between abbreviated laparotomy and definitive abdominal closure, a temporary abdominal closure (TAC) device is applied [15–19]. Recently, specialized TAC devices utilizing active negative pressure peritoneal therapy (ANPPT) have been reported to be associated with improved outcomes by a large, multicenter cohort study and a small, single-center randomized control trial (RCT) [18, 20].

The current standard of care for OA management involves the use of the Barker's Vacuum Pack (BVP) TAC device since all materials are readily available in the operating room [16, 17]. Typically, a nonadherent, manually perforated, polyurethane sheet is placed over the viscera and then covered by either moist surgical towels or gauze. Two Jackson Pratt tubes, connected to variable levels of wall suction, are also used to aid peritoneal fluid drainage [18]. However, variations in BVP are common and lend to the variable efficiency of treatment [19]. Thus, novel TAC devices have been manufactured. The ANPPT dressing used in the study [20, 21] was delivered using the commercial ABThera™ Active Abdominal Therapy System (ABThera Therapy; KCL, an Acelity company, San Antonio, TX). The distinguishing features of this ANPPT are the two polyurethane open cell abdominal foam inserts (although one may be used depending on the size of the abdominal incision) as well as the vacuum tubing set with the T.R.A.C™ Pad coupled with the ABThera negative pressure therapy pump. Bench top testing has suggested that the pressure distribution of the ABThera is superior to the BVP technique, owing to the finger like projections of the open cell foam inserts [19].

Animal models have suggested that ANPPT may more efficiently drain intraperitoneal fluid [22]. Peritoneal fluid (PF), in the setting of peritonitis, has been determined to typically contain many active biomediators at levels much higher than what is observed in the serum [23, 24]. More efficiently draining such PF in animal models fundamentally ameliorated the progression and improved the outcomes of systemic sepsis [22, 24, 25]. Furthermore, preclinical studies have suggested that ANPPT may overcome the immunoparalysis that occurs during the inflammatory intraperitoneal response to septic injury through mediating an anti-infective innate immune response in the face of a compensatory anti-inflammatory response syndrome (CARS) [25]. However, the above-mentioned RCT found no differences in the behaviour of commonly studied leukotrienes between patients randomized to ANPPT or the BVP [20, 26]. Unfortunately, none of the traditional cytokines/acute phase proteins analyzed (Procalcitonin, C-reactive protein, TNF*α*, IL-1*β*, IL-6, IL-10, and IL-12p/70) were predictive of outcome. Thus, we examined the potential role of both plasma and PF HMGB-1 in an OA patient cohort to provide insight into the behaviour and role of HMGB-1 in the critically ill/injured.

## 2. Methods

The Peritoneal VAC trial was a prospective randomized trial involving the random application in the operating room of either a “home-made” Barker's Vacuum Pack (BVP) or the ABThera (ANPPT) in critically ill/injured adults deemed to require OA therapy [20]. The detailed methodology [26] and results [20] have been previously reported; noting there was a marked survival advantage with ANPPT. The study involved attempted collection of plasma and PF samples at 5 specific time points, intraoperative enrolment, followed by 24, 48, 168, and 627 hours after for all surviving patients. Out of the 45 patients enrolled for the original study, only 44 patient samples were analyzed in the current study due to limited sample quantities for one patient. Samples were not available in those patients who died, nor was PF collection available after successful facial closure in surviving patients; thus among the 44 remaining patients there were 319 analyzable samples out of a potential of 450. Patient population demographics of the original Peritoneal VAC trial are reported in Supplemental Table  1 in Supplementary Material available online at https://doi.org/10.1155/2017/6305387.

In addition to a modified processing method devised at the Snyder Translational Laboratory in Critical Care Medicine at the University of Calgary, commercial human HMGB-1 ELISA kits (MyBiosource, San Diego, CA) were combined to allow the detection of HMGB-1 in both patient plasma and PF samples. The analysis of HMGB-1 in human PF is a novel investigation with no prior reports in the literature. The sandwich ELISA kits used had a sensitivity of 19.5 pg/mL, with a detection range of 78–5000 pg/mL. Patient plasma and PF samples were thawed and centrifuged for 10 minutes at 4°C (5000 rpm; Eppendorf centrifuge 5417R). 40–50 *μ* of the supernatants were aliquoted and frozen at −80°C until required. Samples were diluted to 1 : 10 prior to running the ELISA kits as per the manufacturer's instructions. However, high background between sample duplicates required an additional wash cycle during the second washing phase. During all incubation steps (37°C), the ELISA plates were placed on a shaker set at 75 rpm to ensure sufficient mixing of the well contents.

Nonlinear standard curve analysis of HMGB-1 was performed using CurveExpert Professional 2.0.4 for Macintosh. Concentration values were plotted on the *y*-axis, with absorbance (450 nm) values on the *x*-axis. The logistic equation *y* = *a*/(1 + *be*^(−*cx*)^) was used to generate the fitted standard curve.

### 2.1. Statistical Analysis

HMGB-1 levels were log transformed and then summarized using medians and interquartile ranges (IQRs). Whiskers were drawn at the data minima and maxima within 1.5 IQRs. Outliers were plotted separately. Only plasma HMGB-1 levels at baseline (on the first operating day) and at 24 and 48 hours were included in the statistical analysis due to multiple missing samples at late time points (168 and 627 hours after laparotomy). The Wilcoxon-Mann-Whitney test (WMWT) was used to compare survivors and nonsurvivors or ANPPT with BVP cohorts to determine differences in mediator levels, with *p* < 0.05 considered significant.

HMGB-1 levels between plasma and peritoneal fluid samples at 24 and 48 hours were compared using the Wilcoxon Signed Rank test, since the two sample types are dependent. The Spearman correlation coefficient was used to test the assumption that the samples should be paired. To model the predictive ability of baseline HMGB-1 concentration on patient 90-day survival, a simple logistic regression was used; therapy and disease severity at baseline (using APACHE II and SOFA scores) was controlled for when generating the logistic regression. To ensure this approach as appropriate, the linearity assumption was first confirmed by checking the plot between the log odds of survival and plasma HMGB-1 levels. Any nonlinear association would violate the assumption. A *p* value < 0.05 was considered significant. Under the null hypothesis that there is no correlation between the log odds of mortality and HMGB-1 concentration, we would expect the odds ratio for mortality, as calculated by dividing the log odds of mortality by the log odds of survival, to be 1.

All statistical analyses for HMGB-1 median levels and the associated boxplots were done using MATLAB R2014b software for Macintosh. Stata 12 SE software was used to generate the logistic model of baseline HMGB-1 levels and patient outcome.

## 3. Results

### 3.1. HMGB-1 Levels and Survival

**Figure 1:**
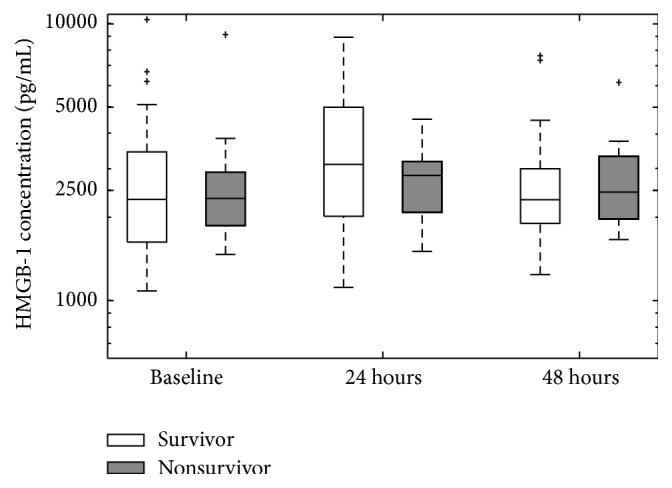
Plasma HMGB-1 levels at enrolment and 24 and 48 hours in relation to survivorship. Boxes represent median and interquartile values, while whiskers represent the maximum and minimum values within 1.5 interquartile ranges (IQRs) of the upper and lower quartile, respectively. Outliers are plotted separately and represented as “+”. No significant differences were observed between survivors and nonsurvivors at all time points (baseline: *p* = 0.84; 24 hours: *p* = 0.54; 48 hours: *p* = 0.37). A total of 44 patients were sampled: 29 survivors and 15 nonsurvivors.

There were no statistical differences between plasma and PF HMGB-1 concentrations at 24 and 48 hours after abdominal laparotomy (Table 1). Thus, only plasma HMGB-1 levels were considered for analysis and reported in the study. For all patients, regardless of TAC allocation, there were no differences in median HMGB-1 plasma concentrations between survivors and nonsurvivors (Figure 1). The levels at baseline enrolment in the operating room for survivors were 2319.81 pg/mL (IQR 1625.33–3442.83 pg/mL), nonsurvivors: 2339.10 pg/mL (IQR 1867.36–2912.64 pg/mL), *p* = 0.84. At 24 hours after laparotomy the levels for survivors were 3070.74 pg/m (IQR 2016.76–4996.18 pg/mL), nonsurvivors: 2831.98 pg/mL (IQR 2081.79–3182.71 pg/mL), *p* = 0.54. At 48 hours after laparotomy the levels for survivors were 2314.07 pg/mL (IQR 1897.73–2994.57 pg/mL), nonsurvivors: 2467.43 pg/mL (IQR 1965.01–3294.88 pg/mL), *p* = 0.37 (Figure 1).

However, individual baseline HMGB-1 levels are not considered in the summarized boxplots; thus intrapatient HMGB-1 trends were normalized to their respective baseline levels and plotted over time (results not shown). Generally, there was a trend for HMGB-1 levels to increase between baseline and 24 hours in both survivors and nonsurvivors, followed by a decrease between 24 and 48 hours. While there were no distinct patterns in HMGB-1 levels that separated survivors from nonsurvivors, there was greater variation in HMGB-1 changes over time for survivors whereas nonsurvivor HMGB-1 levels remained closer to baseline (results not shown).

Overall no significant differences in HMGB-1 levels between survivors and nonsurvivors were identified; however, due to the different therapeutic treatments and underlying conditions of the patients enrolled in the study (septic versus blunt trauma versus perforating trauma) further subgroup analysis was performed. In these subsequent analyses, no significant differences in HMGB-1 levels were observed between patients randomized to different therapeutic interventions (ANPPT or BVP) (Figure 3); however a significant difference in HMGB-1 was observed when comparing septic patients to trauma patients at baseline (Figure 4).

### 3.2. Therapeutic Interventions

**Figure 2:**
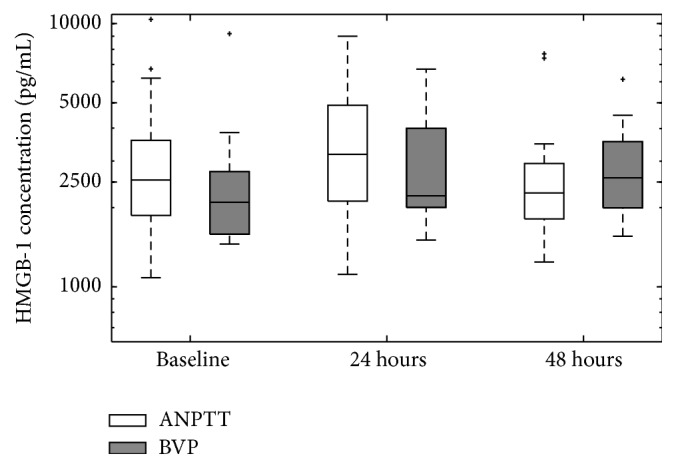
Plasma HMGB-1 levels in relation to TAC dressing applied. Outliers are plotted separately and represented as “+”. 22 patients were in each therapeutic group.

**Figure 3:**
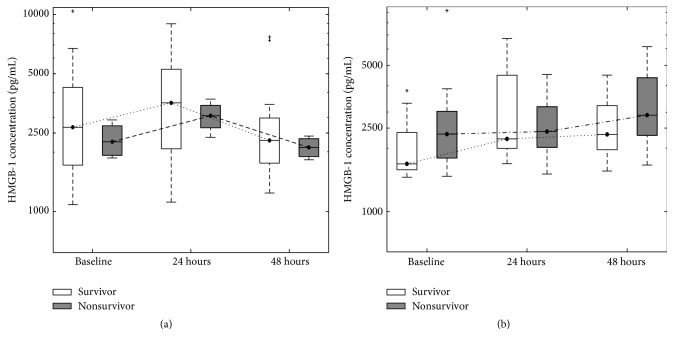
Plasma HMGB-1 levels in relation to TAC applied and survivorship at baseline and at 24 hours and 48 hours after allocation. (a) shows data for the ANPTT allocation (22 patients total: 18 survivors, 4 nonsurvivors). (b) shows data for the BVP allocation (22 patients total: 11 survivors, 11 nonsurvivors). The “+” symbol represents outliers, which were plotted separately.

In subgroup analysis, considering the allocated TAC, no significant differences in HMGB-1 levels were observed between patients randomized to either ANPPT or BVP (Figure 2). Differences in HMGB-1 plasma concentrations between survivors and nonsurvivors for both the ANPPT and BVP groups were not significant at baseline, 24 hours, or 48 hours of therapy (Figure 3). With ANPPT, identical trends were seen for both survivors and nonsurvivors where HMGB-1 levels peaked at 24 hours (survivors: 3556.90 pg/mL, IQR 2078.31–5263.65 pg/mL; nonsurvivors: 3056.19 pg/mL, IQR 2642.14–3439.34 pg/mL; *p* = 1) and then returned to near baseline levels at 48 hours (survivors: 2293.42 pg/mL, IQR 1759.22–2980.51 pg/mL; nonsurvivors: 2111.28 pg/mL, IQR 1895.72–2333.99 pg/mL; *p* = 0.73) (Figure 3(a)). Even though not significant, survivors in the ANPPT group tended to have higher HMGB-1 levels than nonsurvivors at all time points.

There did however appear to be a potential difference in the behaviour of HMGB-1 levels over the time of therapeutic NPPT. Utilizing the BVP therapy, HMGB-1 levels increased from baseline and continued to rise to higher levels at 48 hours for both survivors and nonsurvivors (Figure 3(b)). Within the BVP group, HMGB-1 levels in nonsurvivors appeared to be higher than survivors, a trend that was opposite to the one observed in the ANPPT group. This was especially evident at baseline where HMGB-1 levels were 40% higher in nonsurvivors (*p* = 0.19) (Figure 3(b)).

In patients who were allocated to the ANPPT therapy, the odds ratio for mortality was 0.328 (CI: 0.055–1.941; *p* = 0.157) while adjusting for HMGB-1 concentration and disease severity levels at baseline. Thus, being randomized to the ANPPT group decreased the odds of mortality, since the odds ratio between mortality and survival is less than one; the ANPPT allocation decreased the likelihood of morality by 0.33 times.

### 3.3. Underlying Condition

**Figure 4:**
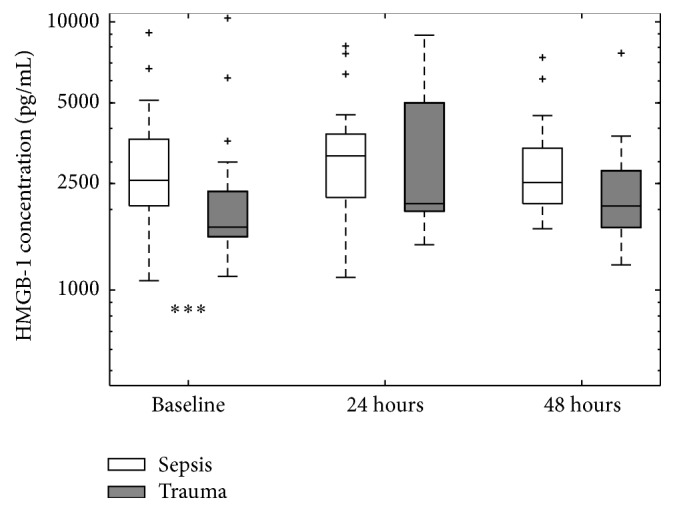
Plasma HMGB-1 levels in contrasting sepsis (*n* = 24) and trauma (*n* = 20) groups. “*∗∗∗*” indicates significant (*p* < 0.05) difference in plasma HMGB-1 median concentration between groups at a particular time point. Outliers were plotted separately and indicated by “+”.

Significantly higher baseline HMGB-1 levels were observed in septic compared to trauma patients (Figure 4). In septic patients HMGB-1 concentrations were higher in survivors than nonsurvivors at all time points (see Online Supplement), although differences were not statistically significant (baseline: *p* = 0.77; 24 hours: *p* = 0.75; 48 hours: *p* = 0.84).

## 4. Discussion

Regardless of overall survival, or survival based on the patient subgroups of therapeutic intervention or underlying condition, there were no significant differences in HMGB-1 levels between survivors and nonsurvivors. There were also no differences in the plasma versus PF levels of HMGB-1 in aggregated patients (Table 1). Thus, in what we believe is the first exploration of Damage-Associated Molecular Patterns in relation to active negative pressure peritoneal therapy, HMGB-1 levels were not obviously explanatory in understanding a potential survival benefit in open abdomen management for critical illness/injury. However, different behaviours of HMGB-1 levels with different TAC management may suggest avenues for future studies exploring the benefits of ANPPT, since the study presented only discrete HMGB-1 median concentrations at isolated time points. What may be of greater interest, to reveal differences between respective subgroups, would be the kinetic change in HMGB-1 over time. Previous studies suggested HMGB-1 levels increase earlier (less than an hour after injury) in trauma or haemorrhagic shock patients than those with sepsis [4, 27, 28]. Findings from both animal models with sepsis and septic patients have shown a delayed elevation of serum HMGB-1 levels [6, 27]. Therefore, those authors speculated HMGB-1 might be involved in the early systemic inflammatory response in injured patients but might also function as a late proinflammatory mediator in septic patients. Our findings demonstrated another kinetic change of plasma HMGB-1 levels in severe septic and injured patients with open abdomen management. Both treatment subgroups have shown slightly higher HMGB-1 levels at 24 hours postoperatively than at the time of laparotomy (Figure 3), which then declined at 48 hours postoperatively. This observation appeared to suggest that the surgical trauma (laparotomy) further boosts the HMGB-1 levels, which is then potentially drained through OA management.

As HMGB-1 levels and behaviour have never before been examined in relationship to OA management, several observations are pertinent. In contradiction to other common inflammatory mediators which are many-fold elevated in PF compared to plasma [23, 24], this was not the case with HMGB-1. As opposed to a biomediator produced/released in response to a stimulatory cascade, HMGB-1 levels result from the more fundamental process of cell death, even before biomediator generation. Interestingly however, the trend observed in most subgroups showed that survivors had higher, though not significant, HMGB-1 levels. These results were somewhat unexpected, since it has been shown in animal models that elevated levels of HMGB-1 are associated with mortality [6] and that treatment with anti-HMGB-1 antibodies improves survival [6]. However, elevated HMGB-1 levels in survivors may be due to the effect of the immune system itself whereby HMGB-1 is passively released by innate response mechanisms such as neutrophil extracellular traps (NETs). These NETs are essentially DNA structures released by neutrophils in an attempt to sequester and destroy pathogens [29]. Since HMGB-1 is a DNA binding protein, it is entangled within these NETs and can passively diffuse into the extracellular environment [5]. HMGB-1 also has antibacterial activity [8]; thus elevated expression of this protein may represent a mechanism used by the immune system to combat infection in addition to being a marker of cellular damage. This may also explain why septic patients had higher HMGB-1 concentrations than trauma patients at all three time points, with a significant difference at baseline (Figure 4). However, our findings were in agreement with previous clinical studies where HMGB-1 levels were not indicative of patient outcome for both septic [7, 27] and trauma [4] populations. In these studies, the findings were more conservative indicating that elevated levels of HMGB-1 might correlate with disease severity and not overall survival.

When compared to other clinical HMGB-1 studies [4, 7, 27], the present study reported lower HMGB-1 levels, and this may be due to the selected detection method. It has been reported that HMGB-1 detection by ELISAs leads to false low or negative results when compared to western blot methods [30–32], and of the referenced studies only Sundén-Cullberg et al. [27] used western blotting to assess HMGB-1. The reported HMGB-1 levels were about 10-fold higher than the present study results. HMGB-1 is a sticky molecule that tends to bind to other molecules to form immune complexes [5, 30], which prevents their detection by ELISA, leading to false low or negative results when compared to western blot methods [30, 31]. Western blotting relies on both linearizing and coating proteins in negative charge in order to determine molecular weight; thus the protein in question is isolated from other molecules that may have been bound to it previously. With ELISAs however, the target protein is left in its endogenous configuration. And in the case of HMGB-1, it may be bound to other molecules such as host IgG antibodies [31], IL-1*β* [32], or other cytokines [32]. The redox state of HMGB-1 might also have an effect on ELISA binding. HMGB-1 has three reducible cysteines whose redox states determine the immunological function of HMGB-1, whether it acts as a cytokine or chemokine or it is inactive [5]. However, to date there has been no reported literature in this regard on ELISA HMGB-1 detection.

It is becoming apparent that HMGB-1 may be a substance that is more important in the chronic phases of severe sepsis than the initial mediator storm of early sepsis. Thus, a limitation of this study may have been only analyzing HMGB-1 levels for the first 48 hours after index surgery. Porcine laboratory studies have demonstrated that PF from septic swine treated with ANPPT may also more effectively stimulate a stronger in vitro reactive oxygen species response compared to no positive pressure therapy [25]. This capacity also peaked earlier, suggesting that ANPPT may have a dynamic effect on the peritoneal microenvironment and may maintain a critical anti-infective innate immune response in the face of a predominate compensatory anti-inflammatory response (CAR) mounted by the host [25]. If ANPPT fundamentally affects the longer-term responses to sepsis, such as CARS or PICS, then continuously improving survival differences may be seen, as were in the Peritoneal VAC trial [20]. Thus, differential clearance of HMGB-1 in mid to late phase resuscitation in critical illness/injury may influence the ultimate survival of these hyperinflammatory patients secondary to the influence of ANPPT. Future studies should thus assess the relative or differential levels of DAMPs, such as HMBG-1, not only in the initial proinflammatory phases of severe sepsis, but also over time into the postinflammatory anergic stages of the process.

When looking at the conditions of the study, it is interesting and potentially worthwhile to note the opposing trends in HMGB-1 time course patterns between the two therapeutic interventions studied. ANPPT has been shown to improve survival after abdominal laparotomy [13, 20], and through the analysis of HMGB-1, we observed that levels peaked at 24 hours and returned to baseline-like levels at 48 hours (Figure 2). However, with the BVT, HMGB-1 levels steadily increased from baseline to 48 hours (Figure 2), which was apparent for both survivors and nonsurvivors (Figure 3(b)). Although the trends are interesting, it must be noted that HMGB-1 levels between both therapeutic groups at each isolated time point were not significant. However, the* kinetic* change of HMGB-1 concentration over time was not analyzed in the current study but may be important in understanding the survival benefit of the ANNPT over the BVP [20]. Thus further study is required, involving an increased number of patients in order to address the potential changes in HMGB-1 levels between 24 and 48 hours in an effort to determine whether therapy plays a role in the observed trends.

## 5. Conclusion

Different levels of HMGB-1 were not found between different treatment cohorts, and thus this representative DAMP did not explain a mortality difference with ANPPT after severe injury or illness. However, differential behaviour of HMGB-1, or other DAMP levels, with ANPPT is a potential mechanism that might be explanatory and should be further explored with future studies targeting a more homogeneous patient cohort with more frequent mediator sampling.

## Supplementary Material

The supplementary material includes the patient demographics of the samples utilized in the study, as well as further plasma HMGB-1 concentration data with respect to TAC allocation, and underlying patient condition; presented either in a table and/or figure.

## Figures and Tables

**Table 1:** Comparison of median plasma and peritoneal fluid HMGB-1 levels with IQR provided in the parentheses. Results based on data available for 44 patients: 29 survivors and 15 nonsurvivors.

	Plasma HMGB-1 (pg/mL)	Peritoneal fluid HMGB-1 (pg/mL)	*p* value
Survivors			
24 hours	3070.74 (2016.76–4996.17)	2835.55 (2180.44–4293.32)	*p* = 0.67
48 hours	2314.07 (1897.73–2994.57)	2236.79 (2037.97–2507.62)	*p* = 0.49
Nonsurvivors			
24 hours	2831.98 (2081.79–3182.71)	2565.58 (2453.06–3266.22)	*p* > 0.99
48 hours	2467.43 (1965.01–3294.878)	2316.53 (2062.03–4322.94)	*p* > 0.99
