# Supplementary material for: High Mobility Group Box-1 Protein and Outcomes in Critically Ill Surgical Patients Requiring Open Abdominal Management
Source: Mediators Inflamm. 2017 Feb 14;2017:6305387. doi: 10.1155/2017/6305387 (PMC5329691; doi:10.1155/2017/6305387)
Supplement: Supplementary file 1 — The supplementary material includes the patient demographics of the samples utilized in the study, as well as further plasma HMGB-1 concentration data with respect to TAC allocation, and underlying patient condition; presented either in a table and/or figure. [file 6305387.f1.pdf]

On-Line Supplements

On-line Supplemental Table 1 — Demographic characteristics of patients enrolled in the Peritoneal VAC trial

Variable	ABThera (n = 23)	Barker's Vacuum Pack (n = 22)	*P*
Age, median (IQR), yr	56 (39–71)	56 (33–68)	0.83
Male sex, n (%)	19 (82.6)	18 (81.8)	>0.99
Abdominal injury, n%	10 (43.5)	11 (50.0)	0.77
Penetrating, n/total n (%)	6/10 (60.0)	2/11 (18.2)	0.08
Mechanism of injury, no./total no. (%)			
Motor vehicle collision	2/10 (20.0)	6/11 (54.5)	0.14
Pedestrian vs motor vehicle	0/10 (0)	1/11 (9.1)	1.0
Gunshot wound	3/10 (30.0)	2/11 (18.2)	0.64
Stab wound	3/10 (30.0)	0/11 (0)	0.09
Other	2/10 (20.0)	2/11 (18.2)	1.0
Injury Severity Scale score, median (IQR)*	23 (18–34)	34 (22–34)	0.32
Revised Trauma score, median (IQR)†	5.4 (1.8–7.8)	5.3 (1.0–6.4)	0.45
Abbreviated Injury Scale scoring, mean ± SD‡			
Head and neck	4.0 ± 0	3.25 ± 0.5	0.10
Thorax	2.9 ± 1.0	3.7 ± 0.9	0.08
Abdomen	3.6 ± 0.7	3.1 ± 1.2	0.20
Extremities/pelvis	3.0 ± 0	2.667 ± 0.5	0.50
APACHE-II score, mean ± SD§	22.5 ± 8.9	26.6 ± 11.9	0.20
SOFA score, mean ± SD¶	7.7 ± 3.9	9.4 (4.7)	0.19
Charlson Comorbidity Index score, median (IQR)‖	3 (1–6)	2 (0–3)	0.04
Worst physiologic measurements before randomization, median (IQR)			
Systolic blood pressure, mm Hg	90 (80–108)	84 (60–91)	0.27
Temperature (injured patients), °C	36 (35.8–36.3)	35.3 (33.5–36)	0.15
Temperature (sepsis patients), °C	36.2 (36–38.1)	37.6 (36.6–38.6)	0.13
pH	7.2 (7.1–7.3)	7.2 (7.1–7.2)	0.15
Lactate, mmol/L	3.6 (1.8–6.6)	6.3 (2.3–10.3)	0.06
Base deficit, mmol/L	10 (7–17)	12 (9.5–17.5)	0.40
INR	1.5 (1.1–1.8)	1.5 (21.2–1.7)	0.84
Fluid administration before randomization, median (IQR)			
PRBC, units (n = 21 injured patients)	10 (3–20)	12 (6–22)	0.30
FFP, units (n = 21 injured patients)	2.5 (2–8)	3 (0–6)	0.54
PRBC/FFP ratio (n = 21 injured patients)	4:1	4:1	
Crystalloid, L	2.5 (1–3.3)	3.2 (2–4.5)	0.21
Patient location before OR admission, n (%)			
Emergency department	12 (52.2)	15 (68.2)	0.73
Hospital ward	5 (21.7)	2 (9.1)	0.41
Intensive care unit	6 (26.1)	5 (22.7)	1.0
Vasopressors required before randomization, n (%)	16 (69.6)	16 (76.2)	0.44
Hours from injury to laparotomy, median (IQR)	2 (2–5)	5 (2–7)	0.38
Hours from sepsis diagnosis to laparotomy, median (IQR)	10 (5–12)	10 (5–10)	0.74

*Values for the Injury Severity score ranged from 0 to 75. Higher values indicate more severe injury.
†Values for the Revised Trauma score range from 0 to 7.84. Higher values indicate greater survival probability.
‡Scores on the Abbreviated Injury Scale range from 1 to 6. Higher values indicate more severe injury.
§Scores on the APACHE-II scale range from 0 to 71. Higher values indicate greater illness severity.
¶Scores on the SOFA scale range from 0 to 24. Higher values indicate greater illness severity.
‖Scores on the Charlson Comorbidity Index range from 0 to 6. Higher scores indicate a lower survival probability.
APACHE indicates Acute Physiology and Chronic Health Evaluation; FFP, fresh frozen plasma; INR, international normalized ratio; OR, operating room; PRBC, packed red blood cells; SOFA, Sequential Organ Failure Assessment.

Reproduced from Kirkpatrick et., Active Negative Pressure Peritoneal Therapy After Abbreviated Laparotomy: The Intraperitoneal Vacuum Randomized Controlled Trial, Ann Surg 262(1), July 2015, p 38–46 (Open Access)

On-line supplement table 2 HMGB-1 levels between different TAC groups

	ANPPT HMGB-1 concentration (pg/mL)	BVP HMGB-1 concentration (pg/mL)	p-value
**Baseline**	2545.64 (1867.36 - 3601.18375)	2084.44 (1582.71 - 2723.94)	p = 0.17
**24 hours**	3182.71 (2114.17 - 4894.15)	2217.10 (2001.33 - 3989.70)	p = 0.30
**48 hours**	2272.94 (1811.20 - 2938.98)	2591.89 (1994.90 - 3552.63)	p = 0.42

On-line supplement table 3 HMGB-1 levels for septic and trauma patient groups

	Sepsis HMGB-1 concentration (pg/mL)	Trauma HMGB-1 concentration (pg/mL)	p-value
**Baseline**	2567.78 (2058.57 - 3651.07)	1718.34 (1580.73 - 2334.22)	p = 0.02***
**24 hours**	3173.53 (2217.10 - 3827.10)	2101.20 (1972.91 - 4996.18)	p = 0.38
**48 hours**	2522.76 (2100.61 - 3387.28)	2060.40 (1709.61 - 2791.01)	p = 0.06

On-line Supplement 4 Differential Behaviour of HMGB-1 plasma levels in relation to underlying patient condition

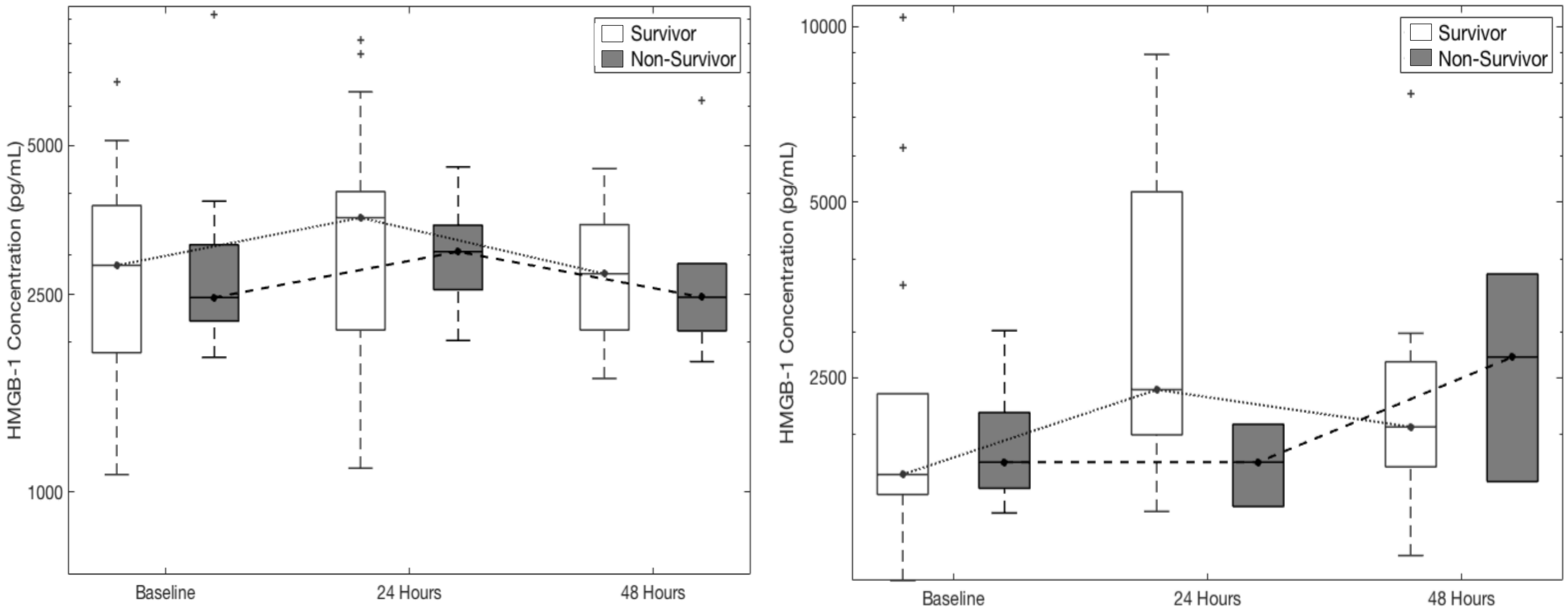

Differential behaviour of HMGB-1 plasma levels between survivors and non-survivors in relation to underlying patient condition measured at baseline, 24 and 48 hours after re-laparotomy. **a)** Septic patient group (15 survivors; 9 non-survivors). **b)** Trauma patient group (14 survivors; 6 non-survivors)
